# Central regulation of food intake, body weight, energy expenditure, and glucose homeostasis

**DOI:** 10.3389/fnins.2014.00384

**Published:** 2014-12-03

**Authors:** Michael M. Scott, Yong Xu, Carol F. Elias, Kevin W. Williams

**Affiliations:** ^1^Pharmacology, University of Virginia School of MedicineCharlottesville, VA, USA; ^2^Department of Pediatrics, Children's Nutrition Research Center, Baylor College of MedicineHouston, TX, USA; ^3^Department of Molecular and Integrative Physiology, Obstetrics, and Gynecology, The University of Michigan Medical SchoolAnn Arbor, MI, USA; ^4^Division of Hypothalamic Research, Departments of Internal Medicine and Neuroscience, The University of Texas Southwestern Medical CenterDallas, TX, USA

**Keywords:** neurotransmission, glucose, resistance, obesity, diabetes, energy expenditure, metabolism

In 1986, only 7 of the 50 states in the US had obesity rates above 10%; whereas in 2010 all 50 states had obesity rates above 10% and 13 states had obesity rates above 30% (C. Centers for Disease, and Prevention, 2010). This is not a problem restricted to the United States, as obesity around the world has doubled since 1980 (Finucane et al., 2011). The burgeoning epidemic of obesity is highlighted by the fact that obesity underlies the development of multiple co-morbidities including diabetes, abnormal cholesterol levels, atherosclerosis, hypertension, heart disease, stroke, reproductive disorders, and numerous types of cancers (Calle et al., 2003; Haslam and James, 2005; Reeves et al., 2007; Danaei et al., 2011; Farzadfar et al., 2011). It is troubling that current strategies for the treatment of obesity and type 2 diabetes mellitus are not optimally effective, and even multiple drug combinations often fail to normalize glycaemia and body weight in a sustained manner in the majority of treated subjects. Thus, understanding how energy and glucose homeostasis is maintained is of upmost importance in order to develop strategies for effective treatments of obesity and diabetes.

In this issue, we have assembled multidisciplinary specialists to provide up-to-date reviews on the recent advances and achievements in the field as well as primary research articles contributing to a greater understanding of obesity and diabetes.

In the initial article, Boychuk et al. (2013) described their original observations that dexamethasone induces a biphasic synaptic responses in feeding-related pre-autonomic neurons in the paraventricular nucleus of the hypothalamus. The authors further demonstrated that the initial increase is mediated by transient receptors potential vanilloid type 1/4 (TRPV1/4), while the subsequent decrease is mediated by cannabinoid receptor 1.

Following the article by Boychuk et al., Tsou and Bence (2012) discuss the current evidence implicating protein tyrosine phosphatases (PTPs) as central regulators of metabolism. The role of a number of PTPs is discussed and specifically their interactions with the neuronal leptin and insulin signaling pathways are highlighted.

This discussion of phosphatases is complemented by the review from Schneeberger and Claret (2012), as they highlight the function of central AMP-activated protein kinase (AMPK) in appetite, thermogenesis, and peripheral glucose metabolism. The authors also discuss the diversity of mechanisms by which hypothalamic AMPK regulates energy homeostasis.

While many of the review articles are focused on the role of proteins and circuits within the hypothalamus in the regulation of metabolic homeostasis, several articles also discuss the importance of the hindbrain circuitry in this regard. In particular, Andresen et al. (2012) nicely summarized current knowledge about the complex neurocircuitry within the hindbrain nucleus of the solitary tract (NTS). The discussion supports a notion that the NTS coordinates major homeostatic reflex pathways to regulate multiple organ systems from gastrointestinal to cardiopulmonary functions.

Maniscalco et al. (2012) also focused on the NTS region in their review, with special emphasis on two distinct populations of neurons which express prolactin-releasing peptide and glucagon-like peptide 1, respectively. Evidence was reviewed to support the view that (i) these neurons contribute importantly to satiation and stress-induced hypophagia by modulating the activity of caudal brainstem circuits that control food intake, (ii) these NTS neurons also engages hypothalamic and limbic forebrain networks that drive parallel behavioral and endocrine functions related to food intake and homeostatic challenge, and (iii) neurons of the NTS modulate conditioned and motivational aspects of food intake.

Focusing again on hypothalamic circuitry, Choi et al. (2013) review data describing the function of the ventral medial nucleus of the hypothalamus (VMH) in homeostatic regulation. The discussion starts from the identification of steroidogenic factor-1 (SF-1) as a marker of the VMH, and then focuses on the emerging homeostatic roles of the SF-1 neurons in the VMH discovered through the use of genetic models; particularly highlighting the control of energy and glucose homeostasis.

Liu et al. (2012) focused on another hypothalamic neuronal population, agouti-related protein-expressing neurons (AgRP neurons), which are both required and sufficient for feeding control. This review was based on recent studies using mouse genetics, novel optogenetics, and designer receptor exclusively activated by designer drugs, and summarized current understanding about AgRP neuron-mediated control of feeding circuits with a focus on the role of neurotransmitters.

Arble et al. (Arble and Sandoval, 2013) provided an elegant and comprehensive summary on the role of the central nervous system (CNS) in regulating postprandial glucose levels. They first highlighted recent evidence to support that the CNS receives and integrates information from afferent neurons, circulating hormones, and postprandially generated nutrients to subsequently direct changes in glucose output by the liver and glucose uptake by peripheral tissues. Further, extensive discussion focuses on the effects of high-fat diet feeding and circadian rhythms on CNS-regulating glucose homeostasis.

Amitani et al. (2013) summarize data regarding the ability of leptin to modulate glucose homeostasis. The authors discuss how leptin acts through specific intracellular signaling cascades in defined populations of neurons while also discussing potential mechanisms of leptin resistance. The literature overview discusses actions of leptin both in the hypothalamus and in neurons that innervate the periphery directly to regulate glucose homeostasis. The review concludes by discussing genetic approaches to manipulating leptin action in the brain and the state of leptin therapy in the clinic.

Donovan and Tecott (2013) discuss the role of serotonin in the regulation of energy balance. The authors provide a thorough summary of research describing how serotonin and drugs targeting the serotonin system (fen-Phen) can affect body weight homeostasis highlighting both the conclusions and controversies associated with the data. The authors also discuss the role of peripheral serotonin on the enteric nervous system and on glucose and lipid metabolism.

Mercer et al. (2013) provide a focused and thorough description of hypothalamic POMC neuron function. The review summarizes the extent of our knowledge on the numerous neuronal populations that innervate POMC cells and the role of serotonin in modulating POMC neuron function, while also highlighting a reciprocal interaction between POMC neurons and NPY/AGRP neurons. The authors also include a discussion on the organization of the POMC neuronal output from the hypothalamus and the data supporting the hypothesis that POMC neurons can be involved in 3 basic circuits, responding to leptin, insulin or serotonin. Finally, the authors discuss genetic approaches involving optogenetics and chemogenetics that are being used to manipulate POMC neurons to better understand their physiological function.

The review from Sohn (2013) discusses mechanisms involved in regulating food intake and metabolic homeostasis from a neurophysiological perspective. The author includes a discussion on various conductances (K_ATP_, Girk, and TRPC) involved in the regulation of several hypothalamic and extra-hypothalamic cell populations involved in energy expenditure as well as glucose sensing/metabolism. The author also summarizes current data describing the role of neurotransmitters (gabaergic and glutamatergic inotropic neurotransmission) in the regulation of hypothalamic and hindbrain neuron activity.

Vanevski et al. (Vanevski and Xu, 2013) discuss the role of brain derived neurotrophic factor (BDNF) signaling in the control of body weight homeostasis. The authors summarize experimental evidence in both animal models and in humans linking dysfunctional BDNF signaling to a perturbation in energy homeostasis. In particular, the authors review studies describing the effect of BDNF in rodent models on food intake through actions in the hypothalamus, primarily in the arcuate nucleus and the ventral medial hypothalamic nucleus. They also discuss the important sites of BDNF production and sites of action within the brain that have been shown to be involved in the regulation of energy homeostasis while also briefly discussing mechanisms of BDNF regulation of glucose homeostasis. Finally, the authors summarize findings demonstrating an interaction between BDNF and other signaling molecules such as leptin, CCK and the melanocortin system in both the hypothalamus and hindbrain.

Khan (2013), presents a unique discussion of methods used to dissect the neuroanatomical pathways involved in the control of feeding behavior in rodents. The author reviews brain microinjection methods, describing both chemical and genetic approaches used to manipulate selected populations of neurons within the brain. Microinjection methods and data are also reviewed from experiments involving both chemical approaches and genetic methods such as those that involve the use of RNAi, optogenetics, and engineered viruses in the characterization of circuits controlling food intake. The author proposes a model of how to integrate data obtained from these diverse methods to gain a better understanding of how specific neuronal circuits affect feeding behavior.

The final review by Udit and Gautron (2013) focuses on the hindbrain, as they review experiments describing the molecular anatomy of autonomic neurons conveying information between the brainstem-spinal cord and the gut. The authors spend the majority of the review describing the latest genetic approaches used in the investigation of this circuit, highlighting their uses, advantages and limitations. The authors also discuss how gut-brain communication is potentially altered in the setting of metabolic disease and how approaches described in the review could be used to better understand any underlying neuronal dysfunction of the gut brain axis.

The aforementioned articles highlight that over the past century prevalent models of energy and glucose homeostasis have been developed from a better understanding of the neural circuits that, when perturbed, lead to the development of obesity and diabetes. Furthermore, these data also demonstrate how the neuronal processes involved in energy and glucose homeostasis also impinge on numerous CNS functions, including the regulation of autonomic outflow and other behaviors. Undoubtedly, utilizing combinatorial research strategies highlighted in these articles will be key to understanding and uncovering potential therapeutics in the treatment of endocrine disorders including obesity and diabetes.

## Conflict of interest statement

The authors declare that the research was conducted in the absence of any commercial or financial relationships that could be construed as a potential conflict of interest.
